# Expansion Culture of Human Pluripotent Stem Cells and Production of Cardiomyocytes

**DOI:** 10.3390/bioengineering6020048

**Published:** 2019-05-24

**Authors:** Minh Nguyen Tuyet Le, Kouichi Hasegawa

**Affiliations:** Institute for Integrated Cell-Material Sciences (iCeMS), Institute for Advanced Study, Kyoto University, Kyoto 606-8501, Japan; le.minhnguyentuyet.8m@kyoto-u.ac.jp

**Keywords:** human pluripotent stem cells (hPSCs), expansion culture, cardiomyocyte differentiation

## Abstract

Transplantation of human pluripotent stem cell (hPSCs)-derived cardiomyocytes for the treatment of heart failure is a promising therapy. In order to implement this therapy requiring numerous cardiomyocytes, substantial production of hPSCs followed by cardiac differentiation seems practical. Conventional methods of culturing hPSCs involve using a 2D culture monolayer that hinders the expansion of hPSCs, thereby limiting their productivity. Advanced culture of hPSCs in 3D aggregates in the suspension overcomes the limitations of 2D culture and attracts immense attention. Although the hPSC production needs to be suitable for subsequent cardiac differentiation, many studies have independently focused on either expansion of hPSCs or cardiac differentiation protocols. In this review, we summarize the recent approaches to expand hPSCs in combination with cardiomyocyte differentiation. A comparison of various suspension culture methods and future prospects for dynamic culture of hPSCs are discussed in this study. Understanding hPSC characteristics in different models of dynamic culture helps to produce numerous cells that are useful for further clinical applications.

## 1. Introduction

Cardiovascular diseases are the leading cause of mortality worldwide. Angina and myocardial infarction known as heart attack is most frequent and occurs when the blood flow to the heart is obstructed, thereby damaging the heart muscles. Heart muscles cannot proliferate by themselves and their permanent loss results in cardiac injury. In order to restore the impaired cardiac function, engraftment of cardiomyocytes by transplantation is necessary [1,2]; however, a limited number of available donors is an obvious and crucial limitation for the transplantation. Recently, production of cardiomyocytes by differentiation from human pluripotent stem cells (hPSCs) has gained immense attention. hPSCs, including human embryonic stem cells (hESCs) [3] and human induced pluripotent stem cells (hiPSCs) [4], have the capability to differentiate into many cell lineages, such as cardiac progenitors, cardiomyocytes, and endothelial cells. These cells are reported to be necessary for formation of cardiac muscle tissue in vivo. Several studies reported the preclinical research using hPSC-derived cardiomyocytes on animal models [2,5,6,7,8,9], and the cardiology department of Osaka University (Japan) announced as the first research group that applied hiPSC-based therapy for human heart [10]. Cardiomyocytes derived from hPSCs can be transplanted to the patient’s body and also applied as a model for cardiac drug screening and disease modeling. Although mature cardiomyocytes are required to appropriately adapt for drug screening and disease modeling, immature cardiomyocytes are applicable for transplantation since the cardiomyocytes can mature in site after transplantation [11,12]. The transplanted cells improved the cardiac function by secreting certain growth factors and cytokines to induce heart repair [13,14]. In order to provide substantial number of cardiomyocytes for transplantation, a large-scale hPSC production system, which can easily be converted to cardiac differentiation, is required. That means hPSC expansion and cardiac differentiation has to be considered as connected processes for cardiomyocyte production. However, many review articles independently focused either on expansion of hPSCs [15,16] or cardiomyocyte differentiation [17,18] or maturation [19,20]. Although a recent review mentioned two-in-one systems that directly incorporate hPSCs expansion into cardiomyocytes differentiation [21], no studies have referred to these integrated systems in different culture formats of hPSCs. In this review, we summarize the methods for expansion of hPSCs in various culture formats and two-in-one systems for integrated hPSCs expansion and cardiomyocyte differentiation. We include discussing the pros and cons of each model and propose the future prospect toward achieving sustainable and scalable, highly efficient production of cardiomyocytes for use in clinical applications.

## 2. Large Scale Expansion Culture of Human Pluripotent Stem Cells

### 2.1. Adherent Culture of Human Pluripotent Stem Cells

Adherent cultures such as monolayers are made by using protein-coating materials such as fibronectin, vitronectin, and laminin [22,23,24] in order to promote cell adhesion on the treated surfaces of the culture dishes or flasks. Recently, synthetic peptides are developed as a cost-effective replacement for the expensive recombinant proteins [25,26]. Melkoumian et al. reported that the application of synthetic peptide-acrylate surfaces by conjugating peptides derived from active domain of vitronectin, laminin, fibronectin, or bone sialoprotein to acrylate can support self-renewal of hPSCs [25]. Notably, Synthemax (Corning), which is a synthetic surface comprising vitronectin-based peptides, is known to be capable of maintaining hiPSCs in chemically-defined medium in a long-term culture [26]. To further develop cost-effective materials, recent studies have also focused on polymer-based coating [27,28,29,30,31,32]. These polymer-based coating materials revealed the capability of maintaining hPSCs in an undifferentiated state; however, the mechanism underlying cell adhesion by using these polymers has not been elucidated. Future studies to clarify the influence of chemical parameter such as hydrophobicity, hydrophilicity, and stiffness on maintenance of multiple cell lines are recommended prior to commercialization of these products.

The simplest xenobiotic-free (xeno-free) medium for expansion of hPSCs is E8 media, which contains eight components: D-MEM/F-12, human insulin, human transferrin, selenium, ascorbic acid, sodium hydrogen carbonate, human recombinant fibroblast growth factor 2 (FGF-2 or bFGF), and transforming growth factor β1 (TGF-β1) [23]. This medium is commercialized such as TeSR-E8 medium (STEMCELL Technologies) and Essential 8 medium (Thermo Fisher); however, the high cost of using this medium creates a barrier for its further implementation in large-scale production. To develop a cost-effective medium, Yasuda et al. demonstrated that replacement of two growth factors in E8 medium (bFGF and TGF-β1) with chemical compounds, including Azakenpaullone, ID-8, and Tacrolimus, are capable of supporting long-term expansion of hPSCs [33]. This medium will be further optimized for cell growth capacity before being commercialized.

To facilitate cell expansion in a monolayer, a large surface area flask of 175 cm^2^ (T175) and its multilayer format with surface area up to 875 cm^2^ is developed (Corning, Falcon). An active gas ventilation system is included to maintain the CO_2_ concentration in all culture layers. Another modern cell culture system called CompacT SelecT was constructed by Sartorius to have a culture area up to 90 T175 along with 96 and 384-well plates for automated cell-based assay in the subsequent stage. In addition, the application of roller bottles (Integra Bioscience) or CellCube (Corning) is also considered to allow dynamic culture of 2D monolayer model in hPSCs scalable culture. 

Monolayer culture has the advantage of being easily setup and is considered as a standard procedure in all laboratories. This method also produces a homogeneous effect since the cells are uniformly exposed to the medium components. Furthermore, this method allows easy assessment of cell quality by directly observing the cells and colonies under a microscope as well as facilitates medium change. 

Despite its advantages as aforementioned, the 2D culture itself has the limitation of requiring a large surface area and hampers cell harvesting due to enzyme treatment. Moreover, 2D culture does not simulate the in vivo microenvironment. Therefore, replacement of 3D culture can be a suitable approach for large scale production of hPSCs.

### 2.2. Suspension Culture of Human Pluripotent Stem Cells

#### 2.2.1. Application of Microcarriers

hPSCs are anchorage-dependent for survival and proliferation. Applications of cell supports, such as several types of microcarriers, hydrogels, and polymers, have been developed to aid the suspension culture of hPSCs. Microcarriers are supporting matrices for culturing adherent cells with a diameter varying from 10 μm up to 5 mm. Cell culture with microcarrier can increase the production capacity by allowing high density cell proliferation. Microcarriers are applicable for cell culturing in a stirrer bioreactor. Various shapes are available, but a round shape is popular. The size and shape of the microcarrier affects the sedimentation rate of cells in cell-microcarrier complexes. The ideal size for microporous microcarrier is about 100–300 µm in general [34]. Besides, the macroporous microcarrier has a large porous structure that increases the surface area with pore size up to 400 µm. Cells can form a confluent layer around the microporous microcarrier while they are entrapped inside the pores of the macroporous microcarriers. Although the macroporous microcarrier has the advantage of effectively protecting the cells from shear stress in the cell culture using a bioreactor, the cell yield was relatively low [35]. Microcarriers can be made from different materials such as plastic, glass, silica, dextran, or cellulose with the surface being coated by collagen or fibronectin to increase cell adhesion. The application of synthetic peptides for microcarrier surface coatings can help to reduce the cost in hPSC culture [36,37]. Among the popular microcarriers, the dextran-based Cytodex and cellulose-based Cytopore microcarriers, which are developed by Amersham Bioscience (GE Healthcare), are widely applied in culturing several cell lines [38,39,40]. 

While culturing of hPSCs, Phillips et al. succeeded in providing a proof-of-principle to attach hESCs on the polystyrene microcarriers for culturing in a spinner flask [41]. The continuous adherence and removal of cells from the uncoated microcarriers presumably results in reduced cell proliferation at each passage. Application of Matrigel (Corning)-coated [42] and vitronectin-coated microcarriers [43] increased the cell adhesion and enabled long-term culture. Notably, by applying polystyrene microcarriers coated with human vitronectin and E8 medium, Badenes et al. established a cost-effective xeno-free chemically-defined culture system for the suspension culture of hiPSCs [43]. Under the dynamic culture of this study, agitation rates around 30–70 rpm were optimal, which resulted in shear stress at 0.08 to 0.26 Pa. These values of shear stress are considerably lower than the predicted value of other cell types: 0.65 Pa for human embryonic kidney cells [44] and 0.78 Pa for murine embryonic stem cells [45]. Recently, by using the synthetic peptide-acrylate surface microcarriers, Badenes et al. demonstrated a scalable culture of hiPSCs by cell transfer from confluent beads to empty beads in a spinner flask. This method enables the long-term expansion of hiPSCs, which achieved 241-fold expansion in 15 days and yielded 3.3 × 10^8^ cells in 15 mL culture medium [46]. 

The conventional applications of microcarriers require the separation of cells from microcarriers via enzymatic dissociation followed by a filtration step, which causes loss of viable cells. To further develop the applications of microcarriers in cell culture without the filtration step, biodegradable microcarriers are also developed [47,48,49]. Rodrigues et al. reported the utilization of dissolvable microcarriers (Corning), which can assist the cell proliferation in spinner-flasks up to 4-fold after 5 days. For harvesting, a solution containing EDTA and pectinase was used in combination with Accutase (Innovative Cell Technologies) to digest the cell-microcarrier complex into a single cell solution [49]. By using dissolvable microcarriers, a high recovery rate of 92% is obtained compared to 45% in conventional polystyrene microcarriers. 

These studies demonstrated that microcarriers application is suitable for scalable culture of hPSCs in a bioreactor to obtain a substantial number of cells and derivatives for clinical purposes. Nevertheless, the disadvantage of dissociating the cells from microcarriers during cell harvesting can be a barrier for further differentiation applications.

#### 2.2.2. Formation of Carrier-Free 3D Aggregates

Another approach in culturing hPSCs in the suspension culture is to form cell spheres by self-aggregation of hPSCs. Dissociation of cells into colonies [50], and then culturing them in absence of the coating material, results in formation of cell aggregates. To further improve the homogeneity of forming colonies, 3D aggregates of hPSCs can also be achieved by dissociation into single cells and culture in the presence of ROCK inhibitor [51,52,53,54]. In addition, a uniform population of aggregates can be accomplished by seeding the cells into fabricated microwells [54,55]. Cells can be cultured in static suspension at small scale by untreated culture dishes or dynamic suspension at larger scale in spinner flasks or bioreactor with agitation. Cell aggregates should be maintained over several passages in the static culture excluded from shear stress in order to enhance the adaptability to subsequent dynamic cultures [50,56]. By optimizing the agitation rates and concentration of shear protectants, Abbasalizadeh et al. were able to produce 3D size-controlled aggregates at clinically relevant numbers of hPSCs (~2 × 10^9^ cells) in one month [52]. In this study, the frozen stock of suspension-adapted hPSCs for bioreactor was also prepared in advance without depending on adaptation in the static culture. Another group also reports the handy scalable stirred culture system to produce numerous hPSCs (2 × 10^9^ cells) only in 14 days utilizing a ready-to-use and commercially-available 3 L bioreactor (Mobius 3 L Bioreactor, Merck Millipore) [57]. An hPSC culture platform conforming to GMP regulation has been developed by Chen et al. [53]. Furthermore, Wang et al. reported successful scalable expansion of hiPSCs as 3D aggregates in the defined xeno-free E8 medium [58]. Collectively, the future prospect of obtaining numerous cells for transplantation can be accomplished using this method.

Due to strong cell–cell interaction and extracellular matrix secretion of hPSCs [51,59], a compact cell sphere can be achieved, which is independent of the anchorage such as microcarriers. Thus, culture hiPSC as 3D aggregates facilitates cell harvesting and maintains the cell integrity for further differentiation applications. Nevertheless, in most cases, a ROCK inhibitor should be maintained not only during the time of sphere formation but also throughout the culture process to assist the cell’s proliferation in spheres. Long-time exposure to ROCK inhibitor was reported to affect the cell metabolism [60] and chromosomal aberration [61,62]. Furthermore, culturing cells in the presence of ROCK inhibitor reduced the expression of E-cadherin and promoted EMT toward mesendoderm differentiation [63], which may bias the direction of differentiation. These are potential disadvantages for maintaining the pluripotency and safety of hPSCs in a carrier-free suspension culture.

#### 2.2.3. Hydrogels

Recently, application of hydrogel for suspension culture of hPSCs has received immense attention as it assists the cells in 3D organization while enhancing cell protection via encapsulation. Hydrogels are water-swollen polymers, which can be used to encapsulate cells into a 3D model. A highly applicable hydrogel is the thermal-responsive hydrogel, which is in liquid form at a low temperature and is solidified at higher temperature. During seeding, cells are mixed to form a complex with liquid hydrogels at 4 °C. The 3D complexes of cell–hydrogels are generally formed after incubation at 37 °C.

Hydrogels are classified as natural (collagen, fibrin), synthetic (polyethylene, poly acrylamide), and hybrid hydrogels comprising natural and synthetic polymers [64,65]. Hydrogels can be prepared as per the protocol mentioned in few previous studies [66,67]. Unlike the microcarriers that require a coating material to attach the cells on the surface, hydrogels encapsulate hPSCs in 3D organization, so that no coating is required. Moreover, addition of ROCK inhibitor is not necessary [68,69] or required only in few cases to increase the cell proliferation [70]. For cell harvesting, the hydrogel can be removed by changing the temperature to release cells from thermo-responsive hydrogel [70] or via enzymatic treatment to degrade cellulose-based hydrogel [69]. Although hydrogels facilitate cell harvest by simply changing the physical conditions, depending on materials, the difficulty in controlling the size of cell spheres in hydrogel encapsulation may hinder the reproducibility of further differentiation process.

#### 2.2.4. Functional Polymers for Suspension Culture of hiPSCs without Agitation

To facilitate the hPSC expansion in the suspension with size-controlled aggregates, independent from the ROCK inhibitor, Otsuji et al. proposed the application of functional polymers including Gellan gum and methylcellulose [71]. Gellan gum allows the cells or spheres to be suspended in a 3D model without agitation while methylcellulose can protect the cell surface from fusion with other spheres to create a homogenous sphere size to support cell proliferation. Since these functional polymers can maintain spheres in 3D suspension, no agitation is required. Thus, the cells are maximally protected from damage by shear stress caused by agitation compared to the conventional 3D aggregates culture. The viscosity of these polymers was adjusted to a low level in order to unaffect the diffusion of soluble factors and no gelation was detected. For the culture method, cells are initially dissociated into colonies and cultured in ROCK inhibitor-containing medium for the first 24 h. After formation of cell aggregates on Day 1, ROCK inhibitor is withdrawn by changing the medium. For the passage, cells can be easily subcultured mechanically by passing through a 50–70 µm mesh and harvested via centrifugation. The withdrawal of ROCK inhibitors is advantageous in maintaining specific metabolism and facilitates the application of nonbiased differentiation capacity. This group was successful in applying this culture method for large scale cell expansion of hESCs in a 200-mL oxygen-permeable bag, which is specifically designed to improve gas exchange thereby promoting cell growth. Although agitation is not required to keep the cells in suspension, presumably, a gentle mixing is necessary to increase the oxygen aeration for large-scale cell production. Another group also reported successful establishment of a 3D culture system using Gellan gum and methylcellulose for culturing of cancer stem cells [72]. For details of culture method for expansion of hPSCs, please refer to Table 1.

## 3. Application of Human Pluripotent Stem Cells for Cardiomyocyte Differentiation

### 3.1. Cardiomyocyte Differentiation as Monolayer Culture

**Figure 1:**
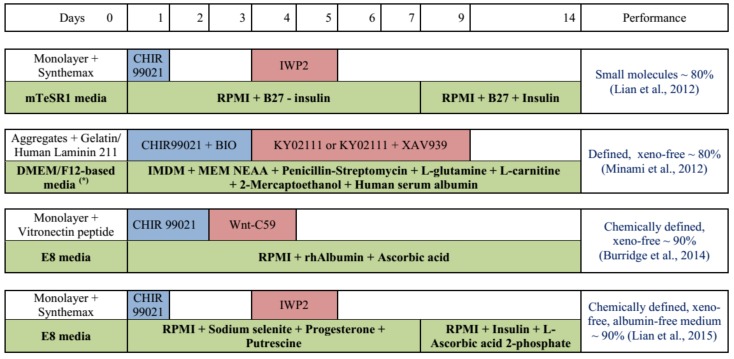
Schematic representation of cardiomyocyte differentiation from hPSCs developed toward xeno-free culture [74,75,76,77]. Abbreviation: ^(*)^ DMEM/F12 + KSR + bFGF + MEM NEAA (non-essential amino acids) + 2-Mercaptoethanol. rhAlbumin: rice-derived recombinant human albumin. The blue and red boxes indicate the period of Wnt activation and inhibition, respectively.

Direct application of hPSCs culture in the monolayer for differentiation is a simple and effective way to induce cardiomyocytes, as all cells can be uniformly exposed to the media components and inducers. 2D culture is applicable for maintenance and proliferation of hPSCs as high and stable expression of pluripotency markers are reported [33,73]. Cardiomyocyte differentiation can be achieved by manipulating the Wnt signaling pathway in a biphasic manner. Specifically, the upregulation of Wnt signaling in the early stages (by small molecules CHIR99021 or BIO) is required to induce differentiation of mesendoderm and mesoderm. Then, downregulation of Wnt signaling by Wnt inhibitors (IWP2, XAV939, or Wnt-C59) is necessary to shape the direction of differentiation into cardiomyocytes. The typical protocol for cardiomyocyte differentiation using small molecules is reported by Lian et al. [74] (Figure 1). This protocol depends on B27 supplement containing bovine albumin serum (BSA), which should be replaced in xeno-free culture for clinical application. Further development toward xeno-free differentiation of cardiomyocytes gaining immense attention [75,76,77]. Minami et al. reported a defined and xeno-free system for cardiomyocyte differentiation independent from B27 by applying the IMDM medium supplemented with MEM nonessential amino acid, penicillin–streptomycin, L-glutamine, L-carnitine, 2-mercaptoethanol, and human serum albumin (HSA) [75]. By comparing the components of B27 supplements with other media beneficial for cardiac differentiation, Burridge et al. found that among several components of B27, only BSA is necessary for cardiomyocyte differentiation. After replacing BSA with HSA and optimizing it, this group reported a chemically defined and xeno-free protocol for cardiomyocyte differentiation with only three components, including RPMI 1640, L-ascorbic acid 2-phosphate, and rice-derived recombinant albumin [76]. In contrast, Lian et al. demonstrated that even BSA is not necessary and only three factors are required for addition in RPMI medium to effectively induce cardiomyocytes, including sodium selenite, progesterone, and putrescine [77]. This protocol introduces an albumin-free system to obtain cardiomyocytes in a cost-effective manner. Further reproduction in 2D culture as well as examining the application of this xeno-free medium in 3D culture is required to improve the efficiency of the differentiation process.

For differentiating the hPSCs into cardiomyocytes at a large scale, Tohyama et al. reported the application of a system combining 4 or 10-layer culture plates with an area surface of 632 cm^2^ [78]. This system yielded 7.2 × 10^8^ hiPSCs in 4-layer and 1.7 × 10^9^ hiPSCs in 10-layer culture plates during the seeding of 1 × 10^6^ hiPSCs per layer. Since the fluctuation of CO_2_ concentration causes mitochondrial dysfunction, which impairs cell proliferation [79], an active gas ventilation system was included to keep the CO_2_ concentration stable in every culture plate. A performance of 66–87% cardiomyocytes was obtained after 10 days of differentiation. In other reports, to increase the purity, a combination step for cardiomyocyte purification using lactate-supplemented media was applied. This process yields 6.2–7.0 × 10^8^ cardiomyocytes in 4-layer culture plates with high efficiency up to 99%. In the 10-layer culture plates, 1.5–2.8 × 10^9^ cells can be achieved, which are sufficient for transplantation in one patient [17,80,81].

Applying cardiomyocytes for drug screening and disease modeling requires not only a large number but also functional mature cells. In attempt to improve the cardiomyocyte maturation, co-differentiation with endothelial cells was approached since the endothelial cells are known to regulate cardiac development in vivo [82,83]. Giacomelli et al. simultaneously differentiated hPSCs into cardiomyocytes and endothelial cells in 2D culture for forming 3D micro cardiac tissue to mimic in vivo development [84]. This co-differentiation enabled yielding cardiac identity endothelial cells, which may be more appropriate for cardiomyocyte maturation than other sources of endothelial cells. In the study, a population of 50% cardiomyocytes and 20% endothelial cells was obtained. Consequently, the cardiomyocytes were enriched up to 80% by labeling with VCAM1 antibody, which is a cell surface adhesion molecule for cardiac cells in the early stage. After enrichment, these cells were mixed with the endothelial cells at a ratio of 85% cardiomyocytes: 15% endothelial cells to form 3D microtissue organization in a 96-well plate. Gene analysis revealed that prolong culture period of these microtissues up to 3 weeks significantly improved the expression of sarcomeric structure markers and Ca^2+^ handling and ion-channel genes. Furthermore, this model reduced the expression of fetal cardiomyocyte genes, suggesting crucial changes toward maturation of cardiomyocytes.

In the monolayer culture, hPSCs were conventionally plated at a high density and were maintained until confluence before differentiation induction was started [85,86,87]. The purpose of plating hPSCs at high density is to take advantage of the secretion signals from high density cells. Relying on cell density effect and its hidden signals may hinder the control of differentiation process. As a new approach, Le et al. proposed to commence differentiation at an initial low cell density to eliminate all the cell-to-cell or cell autonomous signals may present in high-density cells. Once the differentiation procedure is optimized, it enables the control of differentiation process without relying on cell density. That leads higher productivity even differentiation induction is commenced at 1% cell confluency [88].

Despite the monolayer culture of hPSCs providing advantages of evenly exposed cells to all medium components and inducers, this system presented difficulties in cell harvesting and hindered the monitoring of culture conditions. At a later stage, cardiomyocyte maturation by prolonging the culture period [89,90] may not be stably achieved in 2D culture, since the cardiomyocytes tend to detach from the culture surface due to strong contraction. The disadvantage of stability in the 2D culture should be considered for large scale production. Therefore, 3D culture approaches to facilitate scaling up and cell harvesting are good alternatives for large-scale production. 

### 3.2. Cardiomyocyte Differentiation by Suspension Culture Using Microcarriers

Following the expansion of hPSCs using microcarriers, cardiac differentiation can be achieved by simply changing the hPSCs maintenance medium to cardiac differentiation medium [91]. After optimizing the type and concentration of microcarriers, Lecina et al. produced 80% beating aggregates with 20% cardiomyocytes; however, only a low yield of cardiomyocytes was obtained in the spinner culture due to effect of shear stress [38]. In recent years, these authors achieved high efficiency of 83.1% cardiomyocytes after differentiating and purifying them in lactate-supplemented medium in a 500-mL bioreactor scale. Removal of microcarriers by filter through 40–100 µm strainer following enzyme treatment has been proposed [38,42,43]. Utilization of the microcarriers as a matrix for cells is favorable for upscaling to use in the suspension culture except for optimizing the materials, size, shape, and concentration for the microcarriers. Moreover, dissociation of cardiomyocytes by enzyme treatment to detach microcarriers in the terminal stage results in poor survival of cardiomyocytes [18,92]. 

### 3.3. Cardiomyocyte Differentiation by Applying Carrier-Free Cell Aggregates

As an advanced approach, hPSCs can be expanded by forming carrier-free cell aggregates and can be directly applied in cardiomyocyte differentiation. In general, to form cell aggregates, hPSCs are dissociated into single cells by Accutase and cultured in the presence of ROCK inhibitor in a nontreated culture dish, an Erlenmeyer flask, or spinner flask bioreactors. Single cell dissociation enables to control the size of aggregates formed in the later process. Toward industrial production, cell number at the time of induction can be directly adjusted by this method. This approach overcomes the limitation of 2D model since the assessment and adjustment of cell density is easy and is monitored regularly during the culture process. Interestingly, ROCK inhibitor may be useful in priming hPSCs to differentiate into mesendoderm [63], which is the initial step toward cardiomyocyte formation [93]. Hence, addition of ROCK inhibitor during hPSC expansion may be preferred for cardiomyocyte differentiation; however, for versatility in application of cell aggregate culture, addition of ROCK inhibitor may need to be diminished.

Differentiation of hPSC aggregates into cardiomyocytes was reported by several researchers [94,95]. Kempf et al. reported the successful establishment of a suspension culture system in 125 mL Erlenmeyer flasks (20 mL working volume) and 100 mL bioreactor, achieving 60% and 84% cell population expression cardiac Troponin T (cTnT), respectively [94]. Notably, in a similar manner of 3D cardiac differentiation of carrier-free cell aggregates, Chen et al. succeeded in establishing a GMP compliant process to obtain up to 90% cardiomyocytes with a yield of 1.5–2 × 10^9^ cardiomyocytes/L in 1 L spinner flask [95].

According to the Kempf group, a Wnt signal activator (GSK3 inhibitor) CHIR99201 concentration is critical while cell aggregate size is not a prevailing factor for high efficiency of cardiac differentiation across different culture-platforms of 12-well plate, Erlenmeyer and bioreactor. In static 12-well plate, by using NKX2.5-GFP reporter cell line (transgenic cell line carrying green fluorescent protein (GFP) gene under cardiac linage-specific *NKX2.5* gene promotor), this group proved that aggregates of all sizes (around 100–400 µm) and shapes differentiated into contracting GFP^+^ colonies at 7.5 µM CHIR99021. However, at other CHIR99021 concentrations, low GFP expression was observed in all aggregate sizes, suggesting that the aggregate size is not important. In their speculation, the method for 3D aggregates formation in bioreactor is a predominant factor deciding the differentiation efficiency. Aggregates formed by cyclic perfusion feeding are efficient to form cardiomyocytes when batch-feeding is inefficient. Considering this issue, Chen et al. claimed that both CHIR99021 concentration and aggregate size are the dominant factors determining cardiac differentiation in the suspension culture [95]. They noticed that the aggregates obtained on Day 2 after cell inoculation revealed higher cTnT expression, whereas cells on Day 3 presented lower efficiency, thereby suggesting that the larger size of aggregates reduced the differentiation efficiency. They noted that for cardiac differentiation, the optimal concentration of CHIR99021 depends on the aggregate size. Higher CHIR99021 concentrations are needed to effectively promote cardiac differentiation from larger aggregates. Presumably, optimal concentration of CHIR99021 induction for the total number of cell population or depth of cell layers may be the key factor deciding cardiac differentiation efficiency. In addition, the homogeneity in the aggregate size would be required for good reproducibility of differentiation process.

Regarding the homogeneity in cell population of the 2D monolayer culture, several groups noticed that more beating cells can be obtained in the peripheral side of the culture dishes, so-called the rim effect [88,96,97]. Laco et al. demonstrated that the peripheral cells tend to have higher level of cell proliferation, thus promoting cell contraction earlier and stronger than the center cells [98]. Increase in the G1 cell cycle phase and loss in G2/M restrict the cell expansion at the center part of the culture dish [98,99]. Therefore, to enhance the differentiation efficiency in the central part of cell aggregates in 3D culture, small-sized and uniform cell aggregates may be favorable [95].

Nguyen et al. demonstrated that improvement in cardiomyocyte maturation and enrichment can also be obtained by producing a 3D cardiosphere even from 2D differentiated cardiomyocytes [100]. By harvesting the cardiomyocytes on culture plates after 14 days of differentiation and replating into the microwells (Aggrewell 400; StemCell Technologies), a population containing 80–100% beating aggregates can be obtained, with the initial population as little as 10% cardiomyocytes. By applying the same technique of microwell to make aggregates from hPSCs-derived cardiac progenitor, Correia et al. demonstrated that the 3D aggregate culture improves the cardiomyocyte purity and metabolic maturation. Compared to the 2D culture, the 3D culture revealed increased gene expression associated with mitochondrial oxidative phosphorylation and decreased gene expression of glycolysis and lipid synthesis [101]. Collectively, these works proved that 3D aggregates culture indeed results in cardiomyocyte enrichment and maturation.

### 3.4. Cardiomyocyte Differentiation by Applying Hydrogels

A novel approach to directly obtain different culture formats of cardiomyocytes such as microisland, macrotissues, or microsphere by applying hydrogels, is developed [102]. hPSCs are expanded in the 3D model in hydrogel and continuously transferred for cardiac differentiation in 3D model while achieves cardiomyocyte sheet-like structures. As mentioned previously, hydrogel is a hydrophilic polymer which can swell in liquid to form a 3D network and retain its structure until it changes in external physical/chemical conditions [66]. Kerscher’s group used polyethylene glycol (PEG)-fibrinogen hydrogels to encapsulate the hPSCs and form 3D structure for expansion and cardiac differentiation continuously. To form 3D structure from single cell dissociation, ROCK inhibitor was applied only for the first 24 h culture in mTeSR1 medium (StemCell Technologies). Removal of ROCK inhibitor during cell expansion helps to reduce the change in hPSCs metabolism [60]. A population of 75% cardiomyocytes was obtained on Day 20 and could be sustained longer for maturity. T-tubule structure, which is a key component of functional mature cardiomyocytes and has only been seen in postnatal and adult cardiomyocytes, was obtained in this culture model. In this study, only cardiac tissue created on coverslip by micro-island method for drug treatment was reported. As a future prospect, application of several hydrogel models to form macrotissue or microsphere of hPSCs is considered for large scale production of various patterns of cardiomyocytes, which can be used for different biomedical purposes. For details of the culture method for hPSC expansion and cardiomyocyte differentiation, please refer to Table 2.

## 4. Strategies for Cardiomyocyte Maturation

As cardiomyocytes differentiated from hPSCs have similar properties as that of fetal cardiac cell fate [20], these cells should be cultured until they mature in order to be suitable for application in drug screening and disease modeling. Required maturity may be highly variable and depended on target diseases, function, and molecules. Toxicology studies may need most matured cells similar to adult cardiomyocytes. Maturation of cardiomyocytes can be enhanced by co-culturing with endothelial cells [82,83], prolonging the culture time [89,90], controlling the surrounding extracellular matrix [104,105], as well as by applying mechanical and electrical stimulation [106,107,108].

Since cardiomyocytes are subjected to electrical stimulation from the early stage of tubular heart formation, this approach is helpful in increasing cardiomyocyte maturation in terms of improving myofibril ultrasound organization, increasing conduction speed, and promoting both Ca^2+^ handling and electrophysiological properties [106]. Besides, addition of carbon nanofibers to hydrogels to increase the conductivity of hydrogels can help promote cardiomyocyte growth and enhance cardiac function [109,110]. Notably, the application of mechanical stimulation is crucial for cardiomyocyte maturation since the main function of the heart is the mechanical pump that responds to mechanical stimulation throughout its lifetime. By applying cyclic stretch, Mihic et al. improved functional maturation and viability of cardiac tissues [107]. Ruan et al. demonstrated that applying mechanical stimulation revealed improvement in sarcomere alignment and formed stiffer constructs; while addition effect of electrical stimulation did not change the cell morphology but resulted in improved contractility [108]. These data reveal a promising approach of combining multiple stimuli in improving cardiomyocyte maturation. Notably, Shen et al. developed a bioreactor system using cyclic strain and pulsatile flow that can enhance the expression of cardiac-related proteins and genes as well as cardiac ion channel genes [111]. This bioreactor comprises a fluid and an air chamber separated by silicon membrane to ensure a sterile environment. By applying this dynamic culture system, the improvement in SERCA (sarco/endoplasmic reticulum Ca^2+^-ATPase) activity was obtained while maturation level was similar to the primary cardiomyocytes by Raman fingerprint analysis. Combination of dynamic culture and by prolonging the culture time to 20 days exhibited significant change in cardiomyocyte maturation [111]. In the absence of dynamic culture, prolonging culture to several months is required for hPSC-derived cardiomyocytes to achieve the phenotype of adult cardiomyocytes [89]. Thus, dynamic culture plays a pivotal role in promoting cardiomyocyte maturation. 

Several dynamic culture systems were applied for suspension culture of hPSCs and cardiomyocyte differentiation such as the spinner flask (Corning) [95], rotary orbital suspension culture [100], DASGIP, or DASbox Bioreactor System (Eppendorf) [94,112,113]. Application of dynamic culture in the bioreactor to obtain sufficient amount of hPSCs and application for cardiomyocyte differentiation would be an appropriate direction for large-scale production. Dynamic culture has an advantage in not only scaling up but also significantly increasing the functional genes and contractile proteins expression in cardiomyocyte production [114,115,116]. The functional improvement of hPCS-derived cardiomyocytes in a dynamic culture is driven via mTOR signaling pathway [116] and ERK1/2 signaling pathway [114]; however, in a dynamic culture, it is necessary to consider the influence of shear stress on the cell viability and proliferation through physical damage and cell death. Shear stress can also affect the gene expression via mechanotransduction, in which the physical signals are perceived at the periphery of the cell and then converted into biochemical signals in the cell [117]. Although suboptimal values of shear stress resulted in low efficiency of cardiomyocyte differentiation [95,103], appropriate application of shear stress to enhance cell signaling cascade by optimizing agitation rate in 3D culture is an effective approach to improve cardiac differentiation efficiency and maturity [114,118]. 

Besides, extracellular matrix effects, including its composition, alignment, and stiffness, are also important in deciding the cardiomyocyte behavior such as contraction and calcium handling [20,119]. Application of polyacrylamide hydrogel as the stiffness-controllable substrate for cardiomyocytes culture reveals that high degree of maturation can be achieved at intermediate stiffness of 5–10 kPa [120]. Interestingly, Noor et al. recently demonstrated autologous hydrogels produced by processing patient cell-derived extracellular matrix can be applied as a scaffold for cardiomyocyte differentiation [121]. By applying the autologous hydrogels as bioink and 3D printing techniques, they could print vascularized and perfusable heart patches which are completely consistent with the patient’s immunological characteristic. This group of authors really paves the way for engineering hearts in appropriate structure to apply in transplantation, drug screening, and disease modeling.

## 5. Conclusions and Future Perspective

Cardiomyocytes derived from hPSCs are a promising cell source for cell transplantation therapy in heart failures as well as drug discovery and disease modeling for cardiac diseases; however, as discussed in this review, there are several technical barriers to achieve necessary number and quality of cardiomyocytes.

**Figure 2:**
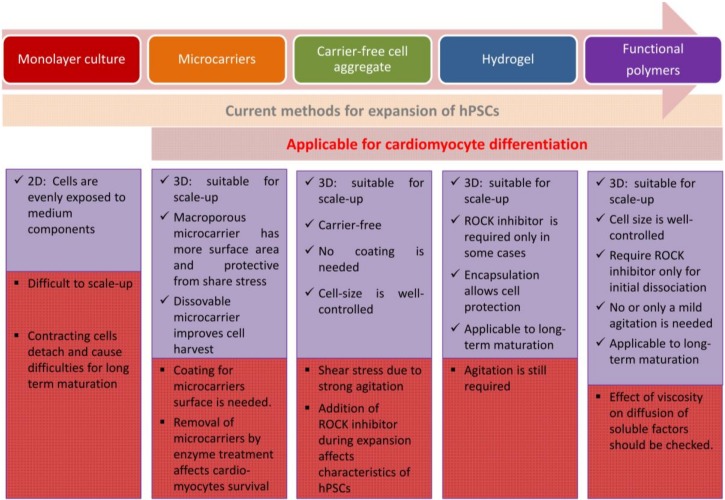
Integrated methods for hPSCs expansion and cardiomyocytes differentiation.

For transplantation, it may be adequate to use numerous immature cardiomyocytes. To date, the efficient methods for cardiomyocyte differentiation from large-scale expansion of hPSCs may include culturing hiPSC as 3D aggregates and hydrogel application to form a 3D structure. For avoiding cellular damage caused by shear stress in the production, encapsulation by hydrogels or macroporous microcarrier will be efficient [35,122]. Applying hiPSC as 3D aggregates independent from the ROCK inhibitor is ideal; however, 3D aggregates along with the ROCK inhibitor is also favorable for cell commitment toward mesendoderm lineage including cardiomyocytes [63,123]. For hPSC expansion and cardiac differentiation in suspension with size-controlled aggregates without depending on carrier and agitation, application of functional polymers as proposed by Otsuji et al. [71] is recommended although the system needs to be improved on oxygen supply in large reactor for industrial-scale cell production. While further studies to examine the effect of viscosity on diffusion of soluble factors are necessary, application of functional polymers for hPSC culture may serve as a future prospect for hPSC expansion at industrial scale and cardiomyocyte differentiation (Figure 2).

In order to improve cardiomyocyte maturation, co-culture with mesenchymal stem cells or exposure to soluble factors from mesenchymal stem cells may be useful as reported by Yoshida et al. [124]. As the future perspective, the first step to enhance cardiomyocyte maturation can be addressed by co-culture with endothelial cells and mesenchymal stem cells. The identification of factors and mechanisms lead advantage in co-culture system and replacement of the endothelial cells and mesenchymal stem cells with the factors may be necessary for large scale cardiomyocyte production. For the second step, prolonging time in dynamic culture using 3D aggregates in suspension can be applied to achieve further maturation. Application of stiffness-controllable polyacrylamide hydrogels as scaffold in the later stage to mimic the stiffness of native cardiomyocytes can be a good consideration to modulate the extracellular matrix for further enhancing cardiac maturation [125]. Although the 3D culture system has not yet been established, application of polymers to control stiffness of the surface such as polyacrylamide [126] or poly(vinyl alcohol-co-vinyl acetate-co-itaconic acid) [127] may be a good approach to enhance the cardiomyocyte maturation in future. 

In this review, we introduced the culture systems for large scale expansion of hPSCs integrated with methods for cardiomyocyte differentiation and further discussed methods for maturation of the cardiomyocytes. Dynamic cell culture system would be the most practical and productive method for large scale production of cardiomyocytes, which are required for transplantation therapy; however, several issues need to be addressed in each method, such as avoiding dependence on ROCK inhibitors in cell aggregates dissociation, requirement of fine tuning of shear stress, purity and maturation, establishment of xeno-free culture system compatible for various hPSC lines, and verification according to GMP regulation. We hope these issues will be resolved promptly and patients will be able to undergo cardiomyocyte transplantation to cure their disease in the near future.

## Figures and Tables

**Table 1:** Suspension culture methods for expansion human pluripotent stem cell (hPSCs).

hPSCs Culture Format	Characteristics	Method	Advantage	Disadvantage	Efficiency	References
**Microcarrier**	Matrigel-coated cellulose microcarriers for human embryonic stem cells (hESCs) culture	▪ Microcarriers were coated with Matrigel and incubated in the culture medium overnight. Cells can be dissociated via mechanical dissociation or enzyme treatment. For spinner flask culture, cells were dissociated mechanically and seeded at 6 ×10^5^ cells/mL in 25–50 mL medium containing 4 mg/mL coated microcarriers with agitation at 15–25 rpm. ▪ Application of mTeSR1 medium revealed higher expression of pluripotency markers than StemPro.	▪ Cells can be stably maintained for 6 months. ▪ Mechanical dissociation by filter through 100-μm mesh or by pipetting.	▪ Matrigel coating is an undefined extracellular matrix, which is not compatible for clinical application.	Achieved 3.5 × 10^6^ cells/mL in 50-mL spinner flask compared to 1.5 × 10^6^ cells/mL in static microcarrier culture and 0.8 × 10^6^ cells/mL in 2D colony cultures	Oh et al., 2009 [42]
**Microcarrier**	A xeno-free subtract (human vitronectin) and chemically defined medium (Essential 8) culture system for human induced pluripotent stem cells (hiPSCs)	▪ Cells were seeded as small clumps using EDTA dissociation method and cultured with Vitronectin coated microcarriers in E8 medium. ▪ Cells were expanded in spinner flask culture with 50 ml working volume. An intermittent stirring of 3 min at 40 rpm every 2 h was applied to promote cell-microcarrier contact.	▪ Reducing production cost in xeno-free system by using E8 medium which lacks albumin.	▪ For harvesting, cells were removed from microcarriers via dissociation and filtration, resulting in cell loss and reduced cell viability.	Achieved 1.4 × 10^6^ cells/mL after 10 days	Badenes et al., 2016 [43]
**Microcarrier**	Dissolvable microcarriers for scalable expansion of hiPSCs under xeno-free conditions	▪ hiPSCs were cultured on dissolvable microcarriers coated with Synthemax in mTeSR1 or TeSR2 medium in a spinner flask with 30 mL working volume. ▪ At the end of culture process, microcarriers were digested by a harvest solution containing EDTA and Pectinase in PBS.	▪ Provide a cost-effective and efficient method for cell harvesting without the need for filtration.	▪ The yield is still lower (or equal) than that of polystyrene microcarriers control.	Achieved maximum 8.81 × 10^5^ cells/mL using dissolvable Synthemax coated microcarriers at high recovery rate of 92%	Rodrigues et al., 2019 [49]
**Carrier-free cell aggregates (3D sphere)**	Suspension culture of hPSCs in static and dynamic culture, using interleukin and bFGF with serum-free media	▪ Spheres were created by incubating cells with collagenase type IV for 20–40 min and splitting mechanically. Cells were resuspended in DMEM medium supplemented with KSR, bFGF and IL6RIL6 in a petri dish. IL6RIL6 is a potent LIF-related cytokine which supports the hPSCs expansion in the suspension culture. ▪ Cells were cultured in static culture in petri dishes for 3–5 passages before being transferred to the dynamic system of Erlenmeyer or spinner flask. This step of culturing in petri dish without shear stress enhanced smooth adaptation into the suspension culture.	▪ Coating is not required. ▪ Facilitate easy cell harvesting and maintain cell integrity.	▪ Depend on IL6RIL6 for maintaining pluripotency. ▪ Large variation of sphere size from 150 to 500 um, with high apoptosis rate of cells located in the center of spheres. ▪ Required long time adaptation in static culture.	25-fold in 10 days	Amit et al., 2010 and 2011 [50,56]
**Carrier-free cell aggregates (3D sphere)**	Sphere culture of hPSCs with single cell dissociation	▪ Feeder cells were removed by collagenase IV; next, single cell dissociation was carried out by suspending the cells in collagenase B solution for 15 min by shaking. ▪ Cells were cultured in mTeSR1 medium with ROCK inhibitor Y-27632 during expansion.	▪ Single cell dissociation allows formation of homogenous aggregates size, which contributes to the control culture at industrial scale.	▪ Continuous exposure to ROCK inhibitor changes the metabolism of hPSCs.	Cell numbers increase by six-fold within 4 days	Olmer et al., 2010 [51]
**Carrier-free cell aggregates (3D sphere)**	A scalable GMP compliant suspension culture system for hESCs	▪ Cells were treated with ROCK inhibitor for 1 h before dissociating into single cells by Accutase and seeded in StemPro medium with 40 ng/ml bFGF. ▪ ROCK inhibitor was supplemented during expansion. ▪ Culture in 6-well plates or 125 mL spinner flasks required agitation by orbital shaking at 70 rpm to prevent cell attachment. ▪ Homogenous aggregate sizes were achieved. Size at Days 2 and 3 were 100–120 and 150–200 μm, respectively.	▪ Provide a scalable, serum-free, and carrier-free suspension culture system that controls industrial production. ▪ All the procedures were performed under c-GMP regulation.	▪ Relying on ROCK inhibitor during expansion may affect the hPSC characteristics.	An average expansion rate of 4-fold can be obtained per passage of 3–4 days	Chen et al., 2012 [53]
**Hydrogel**	Use a thermo-responsive hydrogel to culture hPSCs	▪ Poly(N-isopropylacrylamide)-co-poly(ethylene glycol hydrogel is a thermo-responsive hydrogel, which is liquid at low temperature and becomes solid at high temperature. ▪ Cells were dissociated into single state by Accutase and mixed with the liquid hydrogel in E8 medium at 4 °C, then incubated at 37 °C to form hydrogels and supplemented with warm E8 medium plus ROCK inhibitor. ▪ Cells were harvested and passaged by washing with cold-PBS to liquefy the gel, collecting cells by centrifugation and dissociation by Accutase.	▪ Hydrogel is defined and biocompatible. ▪ Harvest or passaging by simply changing temperature at the range from 4 °C to 37 °C.	▪ Difficult to control sphere size. ▪ Compatibility between medium and hydrogel should be examined case by case. (mTeSR medium is not compatible).	In single cell passage, cell fold increase is 10 after 4 days	Lei et al., 2013 [70]
**Hydrogel**	Apply plant-derived nanofibrillar cellulose (NFC) hydrogel to culture hPSCs	▪ NFC forms hydrogels in aqueous medium with flexible physical property depending on its concentration (hPSCs form 3D spheroids in 0.5wt.% NFC hydrogel, but not in 1wt.%). ▪ Hydrogel stock was diluted in mTeSR1 media and mixed with cell colonies. Cells were sub-cultured via enzymatic removal of cellulose-based hydrogel (ie. cellulase), then dissociation of hPSCs spheres by EDTA and mechanical method prior to mixing with fresh hydrogel.	▪ Removal of hydrogel facilitates differentiation while maintaining 3D cell organization. ▪ ROCK inhibitor is not required.	▪ For enzymatic removal of hydrogel, cellulase requires optimal working temperature of 45–50 °C, which is not suitable for hPSCs. Incubation time with cellulase (at 37 °C) should be extended to 24 h.	N/A	Lou et al., 2014 [69]
**Functional polymers**	Apply two functional polymers to change the viscosity of medium in the culture hPSCs	▪ The two functional polymers include Gellan gum (0.01–0.02%) to keep the cell aggregates floating without the need of agitation and methylcellulose (at 0.3–0.6%) to suppress aggregates from fusing to each other to maintain homogenous size. ▪ The cells were dissociated into large clumps by CTK (a dissociation agent contains Collagenase IV, Trypsin and Knockout serum replacement) or Dispase and cultured in mTeSR medium plus ROCK inhibitor for 24 hours. Cells are passaged by passing through 50 μm nylon mesh filter.	▪ 3D sphere culture is achieved without shear stress effect. Only a minimum agitation is required for gas exchange. ▪ Applicable for culturing in large volume gas-permeable membrane bags.		Seeding at 13.2 × 10^6^ cells/bag, yielded 1.4 × 10^8^ cells/bag which corresponds to a 12.5-fold	Otsuji et al., 2014 [71]

**Table 2:** Two-in-one system for hPSCs expansion and cardiomyocytes (CMs) differentiation.

hPSCs Culture Format	Characteristics	Culture Vessel	Medium for hPSCs Expansion	Yield	Culture Medium and Format for CMs Differentiation	Efficiency of CMs Differentiation	Advantage	Disadvantage	References
Monolayer culture	Use Matrigel-coated multilayer culture plates with active gas ventilation to maintain CO_2_ stable in all culture plates for culture hiPSCs. Metabolic purification for CMs using lactate-supplemented media.	4-layer or 10-layer of 632 cm^2^ culture plates	Modified Stem Fit medium: Stem Fit medium (Ajinomoto, Japan) with amount of most ingredients increased for large-scale culture. Dissociation by Accutase.	Seeding of 1 × 10^6^ hiPSCs per layer yielded 7.2 × 10^8^ hiPSCs in 4-layer and 1.7 × 10^9^ hiPSCs in 10-layer culture plates	RPMI + B27 minus insulin for 7 days, MEMα + 5% FBS for later stage up to Day 12. Inducers CHI99021 and BMP4.	66–87%. After selection, 99% cTnT was obtained. 6.2–7.0 × 10^8^ cells (4-layer) and 1.5–2.8 × 10^9^ cells (10-layer).	High efficiency in generation of pure hiPSCs-CMs since all cells are evenly exposed to purification medium.	Difficult scalability. Difficult cell harvesting.	Tohyama et al., 2017 [78]
Monolayer culture	Perform co-differentiation to CMs and endothelial cells (ECs) from hPSCs in 2D model then forming 3D cardiac microtissue (MT) by mixing CMs and ECs.	N/A	E8/ Vitronectin coat. Dissociation by EDTA 0.5Mm.	N/A	2D monolayer. V-bottom 96 well microplates for microtissue formation. BPEL medium + BMP4 + Activin-A + CHIR99021. Wnt inhibitor XAV939 from Day 3 for CMs and VEGF for ECs differentiation.	Enrichment by VCAM1+ antibody increased CMs from 63% to 85%. The ratio of 85% CMs and 15% ECs presented a good microtissue organization.	Approach by co-differentiation to get cardiac identity-endothelial cells prior to microtissue formation helps promote CMs maturation	This protocol includes several steps and is only applicable for laboratory scale.	Giacomelli et al., 2017 [84]
Microcarriers	Expansion of hESC followed by CMs differentiation in a homogenous process	Ultra low attachment T-25 flask with rocker culture	mTeSR1/ Matrigel coat. Clump dissociation by dispase.	Seeding of 2 × 10^5^ cells/mL yielded 3.74 × 10^6^ cells/mL after 7 days	Microcarrier aggregates. RPMI+B27 minus insulin with CHIR and IWP2.	Yield 2.45 × 10^6^ CM/mL with 65.73% expression of cTnT after 12 days differentiation	Integrate hPSCs expansion and CMs differentiation in a continuous process	CMs were separated from microcarrier by enzymatic dissociation and filter through 40 µm cell strainer	Ting et al., 2014 [103]
Microcarriers	hPSC expansion, differentiation, and purification using microcarriers	500 mL controlled bioreactor	N/A	3.66 × 10^6^ cells/mL after 7 days culture (18-fold increase)	CMs induction medium: N/A. CMs purification was developed using lactate- supplemented medium.	1.33 × 10^6^ CMs/mL with 83.1% expression of cTnT after 23 days culture and purification	A high yield of CMs can be obtained using a large volume bioreactor	Removal of microcarriers before further applying CMs for transplantation, drug screening and disease modeling	Steve Oh et al., 2017 [91]
Carrier-free cell aggregates (3D sphere)	Two methods to form aggregates Batch-feeding: stirring- Controlled aggregates were formed for 48 h. Cyclic perfusion feeding: 24 h after inoculation, stirring was paused for 10 min every 2 h to let cell aggregate.	Static 12-well plate. 125-mL Erlenmey-er (working volume 20 mL). Bioreactor 100 mL.	mTeSR1 with Y-27632. Single cell inoculation by Accutase	60 × 10^6^ cells in 100 mL bioreactor	Matrix-independent aggregates (3D sphere). RPMI+B27 minus insulin with CHIR and IWP2.	In bioreactor, efficiencies are 85%, 54%, 68% (n = 3) after 10 days of differentiation Erlenmeyer ~ 60.4%	Applying carrier-free cell aggregates facilitates cell harvesting compared to microcarriers	Maintenance of cell aggregates required ROCK inhibitor during culture process may change hPSCs metabolome profile.	Kempf et al., 2014 [94]
Carrier-free cell aggregates (3D sphere)	The culture process is defined and standardized in compliance with GMP regulations.	6-well plate; 125, 500, and 1000 mL spinner flasks	StemPro medium with 40 ng/mL bFGF and ROCK inhibitor. Single cell inoculation by Accutase.	Seeding at 2.5 × 10^5^ cells/mL yielded 1 × 10^6^ cells/mL after 3 days	Matrix-independent aggregates (3D sphere). RPMI+B27 minus insulin with CHIR and IWP2.	>90% CM purity after 25 days of differentiation Yield 1.5 to 2 × 10^9^ CM/L	Integrate hPSCs expansion and CMs differentiation in a continuous suspension culture. Suspension culture eliminates the need for coating.	Maintenance of cell aggregates required ROCK inhibitor during culture process may change hPSCs metabolome profile.	Chen et al., 2012;Chen et al., 2015 [95]
Hydrogel	hiPSCs were encapsulated in PEG-fibrinogen hydrogels and differentiated into CMs continuously	Prepare PDMS mold on acrylated glass, put in 6-well plate	mTeSR1 with ROCK inhibitor supplemented for the first 24 h	Cells were seeded at 5.5 × 10^5^ hiPSCs per tissue	3D engineered cardiac tissues (micro island). RPMI+B27 minus insulin with CHIR and IWP2.	75% cTnT positive (day 20). Maturation structural of CMs including tubules can be obtained.	Cardiac tissue can be formed in single seeding step. CMs cultured in 3D hydrogel can be sustained longer for maturity.		Kerscher et al., 2016 [102]
